# Editorial: Spatial Navigation: Memory Mechanisms and Executive Function Interactions

**DOI:** 10.3389/fnhum.2019.00202

**Published:** 2019-06-13

**Authors:** Thackery I. Brown, Elizabeth R. Chrastil

**Affiliations:** ^1^School of Psychology, Georgia Institute of Technology, Atlanta, GA, United States; ^2^Department of Geography, University of California, Santa Barbara, Santa Barbara, CA, United States

**Keywords:** hippocampus, memory, navigation, executive functions, network

Decades of research have emphasized the importance of the medial temporal lobes for spatial navigation and long-term memory. Recent evidence suggests that structures outside of the medial temporal lobes contribute to spatial navigation by providing additional spatial coding and computations relevant for long-term memory, decision-making, and executive function. Together, multiple neural systems may dynamically interact to provide neural architecture that (1) supports dynamic encoding, maintenance, and updating of spatial information and (2) translates convergent spatial and non-spatial information into navigational memories and goal-directed behavior. It is essential that the field pursue mechanistic accounts of how such spatial codes emerge and interact across the brain, bridging theories of spatial navigation, episodic memory, and executive functions.

Recent empirical and theoretical work on these fronts has begun to tackle that very challenge. For example, one way to advance our understanding of the established role of the hippocampus in spatial memory is to explicitly interrogate its position as a node within broader network dynamics. Arnold et al. demonstrated that not only does the hippocampus serve as a network hub, but this functional position changes across encoding and retrieval. Hippocampal network centrality decreased as encoding demands lessened, both as a connector between modules and within the hippocampal neighborhood. Notably, they observed increased hippocampal network connections during mental simulations based on retrieval. Their results indicate a shift in the network dynamics surrounding the hippocampus as encoding demands change, reconfiguring from global integration to localized processing based on the degree of integration of environmental information. These findings connect with recent explorations of the interaction between “semanticized” spatial knowledge (schemas) and episodic navigational memories (van Kesteren et al.). This line of investigation demonstrates that the role of the hippocampus in spatial memory is quite dynamic, such that modulation after one-shot learning depends on the fidelity of prior spatial knowledge of the environment. Such data suggest that spatial schemas can accelerate new learning, while also reducing the computational demands on the hippocampus for subsequent simulations about the environment.

In light of these insights into the dynamics of navigational memory, an important question to ask is how such hippocampal-extrahippocampal interactions relate to memory performance. Sulpizio et al. combined fMRI with stimuli assessing larger-scale vista space knowledge in a real-world environment. They found that hippocampal activity showed neural adaptation for repeated facing directions, suggesting a spatial heading signal. Conversely, scene-responsive cortical areas showed adaptation to distances, but the hippocampus did not. Critically, the strength of spatial position coding in retrosplenial cortex tracked individuals' ratings of their spatial abilities—suggesting that the locus of individual navigation abilities may extend beyond the hippocampus. This observation complements evidence from Burte et al. that directional sense is tied to an extended hippocampal-cortical network. Here, they observed that gray matter volumes in the hippocampus predicted individual sense of direction, whereas functional brain activity spanning frontoparietal regions—including retrosplenial cortex—were involved in comparing heading directions. Izen et al. examined individual abilities in a path integration task using resting state functional connectivity. They found that functional connections between medial temporal areas and both the right frontoparietal executive network and the default mode network were increased in better navigators. Together, these findings highlight the importance of interactions between the hippocampus and extra-hippocampal regions in defining individual navigational abilities.

The role of retrosplenial cortex in human navigation is a fascinating target for continued research, as evidenced by the studies on individual abilities in this special topic. Not only does this region provide clear direction-related signals in navigating rodents that are distinct from hippocampal spatial codes, but there is considerable variability in the anatomical loci and functional associations of retrosplenial activity across human studies. Burles et al. demonstrate that functional heterogeneity in medial parietal cortex, spanning classically-defined retrosplenial cortex and posterior cingulate, is non-trivial for navigation research. Indeed, there appears to be a dorsal-ventral functional gradient surrounding the posterior cingulate. Regions which are commonly labeled as “retrosplenial cortex” in fMRI studies may therefore be more appropriately referred to as distinct subregions that differentially subserve spatial recall (dorsal) and encoding (ventral).

This special topic also drew important attention to prefrontal circuitry and its relationship to navigational performance. For example, Burte et al. observed relationships between orbitofrontal cortex volume and task accuracy in their study; Izen et al. demonstrated that resting state functional connectivity between the medial temporal areas and networks with prominent prefrontal components predict better path integration ability. This work underlines the importance of future research generating a greater understanding of prefrontal-related executive function in spatial cognition.

What are the ramifications of such observations? One answer is they can provide insight into cognitive development across the lifespan. For example, Sneider et al. examined hippocampal and prefrontal brain activity in adolescents during a virtual Morris water maze task. They observed that during adolescence, worse performance during spatial retrieval was associated with greater BOLD activation of angular gyrus and supramarginal gyrus, whereas worse performance during visible platform navigation was associated with less activation of anterior prefrontal cortex. They suggest that in adolescents, less BOLD activation of the frontal pole in worse navigators could be a sign of less effective navigational path planning. Such questions can also be asked in aging populations, where spatial abilities and strategies may regress in the other direction. In particular, Zhong and Moffat's careful survey of the literature indicates that changes in prefrontal function may play a major role in both strategy switching and spatial association learning as we age.

The mechanistic basis for spatial strategy shifts and associative memory are ripe for continued research, even in the canonical sample of healthy college age adults. As reviewed by Goodroe et al., the classic dichotomy of attributing allocentric spatial memory to the hippocampus and egocentric route-based memories to the striatum is not comprehensive enough to include all of the mechanisms needed for many navigation scenarios. For example, some route-based memories may draw on episodic-memory mechanisms and prefrontal control processes, even after much practice. One alternative to describing navigational scenarios based on modularized brain function or spatial reference frame is to adopt a model-based vs. model-free reinforcement learning perspective of task demands. Starrett and Ekstrom offer a complementary examination of such issues, focusing specifically on challenges for distinguishing egocentric and allocentric spatial representations, while introducing a new task—the relative vector discrimination task. This new paradigm may better target the allocentric dimension of spatial representations than established virtual navigation tasks. Such advances in paradigm structure may help researchers resolve how we flexibly acquire, integrate, and draw on different reference frames of our environments. Understanding reference frames and how they relate to other theoretical perspectives such as reinforcement learning is critical for the field. As He and McNamara show, initial headings when experiencing an environment define a reference frame for the space that can influence subsequent learning and spatial updating. Together, these articles are pushing the boundaries of how we understand reference frames.

How “non-spatial” cognitive differences are associated with different behavioral strategies is also a particularly important direction for continued research. For example, although spatial ability is a clear driving factor, predispositions to anxiety, risk aversion, and enjoyment from the act of exploration, can manifest in profound differences in how we choose to traverse our environment. In fact, as Pazzaglia et al. show, latent spatial abilities better predicted route-tracing performance, whereas measures of anxiety, efficacy, and pleasure in exploring (among other personality traits) were more likely to predict shortcut-finding performance. It is intriguing to think about how such relationships interact with the dynamic nature of real world environments. For example, in this special topic, Piccardi et al. examined spatial memory in L'Aquila earthquake-exposed survivors, and suggest that continuous and extreme environmental changes could mean that people need to attend more to navigational space, leading to improvements in topographical learning. The ability to adjust learning and attentional strategies in a dynamic world may be a critical trait related to whether and how effectively people balance exploration and exploitation to maximize learning and navigational efficiency.

One exciting aspect of bringing such a diverse range of scholars together for a special topic such as this is its power to generate new ideas. Convergent and divergent findings in the empirical work, as well as in the literature reviews, compile and underscore key future directions for the field. For example, as highlighted in the extensive review by Herweg and Kahana, behavioral work in humans does not unequivocally support the use of a metric Euclidean map for navigation. Formal models of navigational behavior, which account for environmental scale and complementary learning mechanisms, may help to better understand different navigational strategies. One approach to refining such models could be to study how place- (and concept-) responsive single-cell activity relates to ongoing theta oscillations during both the encoding and retrieval of spatial and non-spatial associations. These temporally-extended and recurring oscillatory signals could complement fMRI work in the grand objective of unifying theories of medial temporal lobe function under the umbrella of mechanisms that relate or discriminate experiences across multiple temporal and spatial scales.

Other opportunities for continued research into extra-hippocampal mechanisms exist in causal/interventionist approaches. As highlighted in Brunye's review (Brunyé), recent advances in functional connectivity analyses have revealed stable functional networks that include both deep subcortical structures and regions on the cortical surface. This finding suggests that the modulation of superficial brain regions such as the inferior parietal lobule and lateral prefrontal cortex may carry powerful downstream consequences for deeper brain systems involved in spatial processing and real-world navigation. Transcranial electrical stimulation has gained popularity as a tool for modulating several aspects of perception and cognition. As we come to understand the parameters underlying effective excitation and disruption protocols with this tool, we may be able to gain causal understanding of the relationships between, for example, neocortical oscillations and spatial computations. Moreover, our growing understanding of functional network profiles within the navigation system may enable the use of such tools to indirectly target subcortical brain regions by altering neuronal activity in distant—yet functionally connected—cortical areas.

Collectively, the articles in this special topic highlight new and exciting directions for the field of spatial navigation. The studies all look beyond the traditional boundaries of spatial navigation research, either by examining functional brain networks, new techniques, individual differences, or establishing connections with personality traits and executive functions. The innovative ideas generated in this special topic provide a wealth of avenues for future research.

## Author Contributions

TB and EC co-wrote all aspects of the manuscript.

### Conflict of Interest Statement

The authors declare that the research was conducted in the absence of any commercial or financial relationships that could be construed as a potential conflict of interest.

